# Tracking submediterranean ecotone shifts under climate change scenarios using marcescent oaks as indicators

**DOI:** 10.1038/s41598-025-10088-w

**Published:** 2025-11-10

**Authors:** Isabel Passos, Carlos Vila-Viçosa, João Gonçalves, Maria Margarida Ribeiro, Albano Figueiredo

**Affiliations:** 1 Research Centre for Natural Resources, Environment and Society, Polytechnic Institute of Castelo Branco, Polytechnic University, Quinta Sra. de Mércules, Castelo Branco, 6001-909 Portugal; 2 Centre of Studies in Geography and Spatial Planning, Department of Geography and Tourism, Colégio de São Jerónimo, University of Coimbra, Coimbra, 3004-530 Portugal; 3 BIOPOLIS Program in Genomics, Biodiversity and Land Planning, CIBIO. Campus de Vairão, Vairão, 4485-661 Portugal; 4 MHNC-UP—Museu de História Natural e da Ciência da Universidade do Porto – Herbário PO, Universidade do Porto. Praça Gomes Teixeira, Porto, 4099-002 Portugal; 5 CIBIO-InBIO (Research Network in Biodiversity and Evolutionary Biology), Campus Agrário de Vairão, Vairão, Portugal; 6 Prometheus—Research Unit in Materials, Energy and Environment for Sustainability, Instituto Politécnico de Viana do Castelo, Viana do Castelo, Portugal; 7 Polytechnic Institute of Castelo Branco, School of Agriculture, Polytechnique University, Quinta Sra. de Mércules, Castelo Branco, 6001-909 Portugal; 8 Forest Research Centre, TERRA Associated Laboratory, Superior Institute of Agronomy, CEF, Lisbon University, Lisbon, Portugal

**Keywords:** Submediterranean, Climate change, Range shift, Marcescent Oaks, Niche modelling, Ensemble forecasting, Biogeography, Ecological modelling, Climate-change impacts, Plant ecology

## Abstract

The submediterranean is an ecotone distributed across southern Europe and north Africa, marking the transition between temperate and mediterranean climates. This is a remarkable climatic and vegetational dynamic area, with major shifts driven by changes in climate since the Late Quaternary. We explore future range shifts of the submediterranean ecotone caused by climate change using marcescent oaks as a proxy, considering their high prevalence in forests under such conditions. Our results confirm the ongoing dynamism of this ecotone, which is likely to expand its range northwards, where marcescent oaks will overlap Eurosiberian forests dominated by deciduous and temperate species. Conversely, in the southwestern Mediterranean, a loss of suitability is predicted, with remaining suitable habitat only at higher altitudes or along coastal regions, promoting scattered refuge suitable areas. To preserve these forests, further research is needed to assess the future ranges of individual species based on high-resolution models, to plan management and restoration strategies within a context of high disturbance by land use.

**Supplementary Information:**

The online version contains supplementary material available at 10.1038/s41598-025-10088-w.

## Introduction

Forests harbor most of the world’s biodiversity and provide a wide range of essential ecosystem services (ES), including carbon storage, soil formation and conservation, timber production, and cultural benefits^1,2^. Despite their critical benefits, native forests worldwide are increasingly threatened by loss, degradation, and fragmentation. These pressures stem primarily from agricultural expansion and other land-use changes driven by human activities, which can be exacerbated by the escalating impacts of climate change. Due to these challenges, forest conservation and restoration became a crucial issue, and effective measures and governance to mitigate widespread threats and risks are needed^3,4^. To increase long-term chances of success^5^ and ES provisioning enhancement, these measures must incorporate data, knowledge, and spatially explicit projections about the future climatic suitability of species and communities^6,7^. Forests might play a central role in climate change mitigation, but climate change itself may threaten them in many regions, compromising their capacity to provide such mitigation effect, especially in the southern Europe submediterranean ecotone region.

Southern Europe crisscrosses two major Biogeographic Regions: (1) the Eurosiberian, and (2) the Mediterranean. The former presents a climate characterized by cool winters and mild rainy summers, with lower seasonality, while the latter typically presents hot and dry summers^8^. The transition between these two regions is gradual rather than abrupt, representing a longstanding debate and concern among biogeographers seeking to unravel its complexities and implications^9^. This transitional area typically presents a subtype of the temperate macrobioclimate, known as the submediterranean, which behaves as an ecotone. It is mostly distributed across the Southern European Peninsulas, being characterized by milder summers fed by additional amounts of rainfall^10,11^compared to the surrounding areas with Mediterranean climatic pattern. Marking the transition between the deciduous forests of the temperate climate, and evergreen sclerophyllous forests adapted to the Mediterranean climate^12–14^the submediterranean ecotone is characterized by the domain of potential semi-deciduous and marcescent forests, dominated mainly by oaks (*Quercus* L.)^12,13^. Marcescence, i.e., the absence of leaf abscission and consequent fall during autumn and winter^13^is considered an adaptation to submediterranean climate^15^where both summer drought and winter frost are common^12^. This adaptation helps protect leaf buds from desiccation in summer and frost in winter^13^. It also serves as a reservoir for unique and endemic species^13,14,16^as well as species from warm-temperate and even subtropical zones^12,15^presenting high biodiversity, a common feature in ecotone areas^17^.

Throughout Earth’s history, the Mediterranean Basin climate has undergone numerous changes^18,19^with the submediterranean areas experiencing considerable climatic and vegetational dynamics, especially since the Late Quaternary^11^. But the rapid pace of modern climate change^19^ may exceed semi-deciduous and marcescent forests’ ability to adapt or colonize new areas^18,19^. It is unknown how this ecotone and the species within will endure under future climate change scenarios, with the expected changes in precipitation cycles, temperature rise, and extreme climatic events, particularly in the Mediterranean region^20^. Temperatures are anticipated to increase 20% more than the global average, especially during summer, when warming can potentially reach up to 50% higher than the global average^20^. At the same time, precipitation is expected to decline, increasing dryness^20,21^. Given the transitory nature of the submediterranean ecotone, changes in environmental conditions can translate into significant adjustments in the structure and composition of plant communities in these ecotones^17^promoting impacts on ecosystems and on their capacity to provide benefits^13^.

Changes in precipitation time-space patterns and minimum temperature were recently determined as the main driver of past range shifts in submediterranean marcescent forests in the Iberian Peninsula since the Last Glacial Maximum(~ 21 kya)^11^. Thus, the predicted increase of drought intensity, frequency, and duration by future climate projections will likely impact the distribution of the submediterranean forests^13^. In some areas, these forests may be partially replaced by sclerophyll woodlands within their current range^16^. This work builds upon these efforts to reconstruct past dynamics of submediterranean marcescent oak forests during the Late Quaternary^11^. By complementing paleodistribution models with future projections under climate change, we trace the biogeographic continuum of this ecotone across time, from glacial refugia to potential future refuges and expansion zones. This approach provides a comprehensive understanding of the drivers shaping marcescent oak distributions and the resulting implications for forest conservation.

Species Distribution Models (SDM) are widely used in species conservation and management planning, habitat restoration, to identify suitable sites for protected areas^22–24^and for assessing climate change impacts on species range^25^. Spatial models can be prepared to predict past, present, and future species distributions, supporting the assessment of potential impacts on potential range shifts^24^. Depicting how the distribution of marcescent species may change under projected climate conditions is crucial for anticipating the future of these forests^11^. It is necessary to understand how these range shifts will affect or interact with forest conservation management across the southern European and circummediterranean regions to inform forest managers and anticipate conservation actions^14^.

To predict future range shifts of submediterranean oak forests under climate change scenarios in the western Mediterranean region, we used four marcescent oak species, emblematic of submediterranean conditions, as a proxy: the Algerian oak (*Quercus canariensis* Willd.), the Portuguese oak (*Quercus faginea* Lam.), the Downy oak (*Quercus pubescens* Willd.), and the Pyrenean oak (*Quercus pyrenaica* Willd.). By addressing this gap in vegetation and biogeographic research, we provide critical information on the current submediterranean areas of the western Palearctic realm and anticipate the effect of climate change. Our specific objectives are:

- Predict the current distribution of the submediterranean ecotone in the western Mediterranean region and identify the key climatic predictors that shapes it;
- Project the distribution of the submediterranean ecotone under future climate scenarios;
- Quantify the range shifts of submediterranean ecotone and discuss the biogeographic outreach of these results;
- Identify submediterranean areas with higher susceptibility to future climate changes, and putative turnover in dominant oak species.

## Results

### Models’ performance and contribution of environmental variables

The ensemble/combined model has TSS and AUC values of 0.873 and 0.985, respectively, indicating excellent performance (Table 1). Sensitivity and specificity scores were above 90, highlighting a well-balanced model, capable of accurately predicting known presences (test samples) and pseudo-absences.

**Table 1:** Evaluation scores for the ensemble model, cutoff, sensitivity (also called true positive rate or recall), specificity (also defined as the true negative rate), considering two different metrics: TSS (true skill statistic) and AUC of ROC (area under the receiver operating curve). The number of species records presented is computed after removing duplicates.

Evaluation metric	Evaluation scores	Cutoff	Sensitivity	Specificity	Number of species records
TSS	0.873	584	94.7	92.6	8342
ROC	0.985	591	94.7	92.7	

The results indicated that bioclimatic factors were the primary determinants of the distribution of potentially suitable areas for the studied marcescent oaks (Supplementary Fig. 1). In contrast, topographic and soil-related factors were secondary in explaining species’ current distribution. Among the bioclimatic variables, annual precipitation (Bio12) emerged as the most significant contributor, followed by temperature seasonality (Bio04) and the minimum temperature of the coldest month (Bio06). Other factors, including isothermality (Bio03), precipitation seasonality (Bio15), and precipitation during the warmest quarter (Bio18), made smaller yet comparable contributions to the model.

**Fig. 1:**
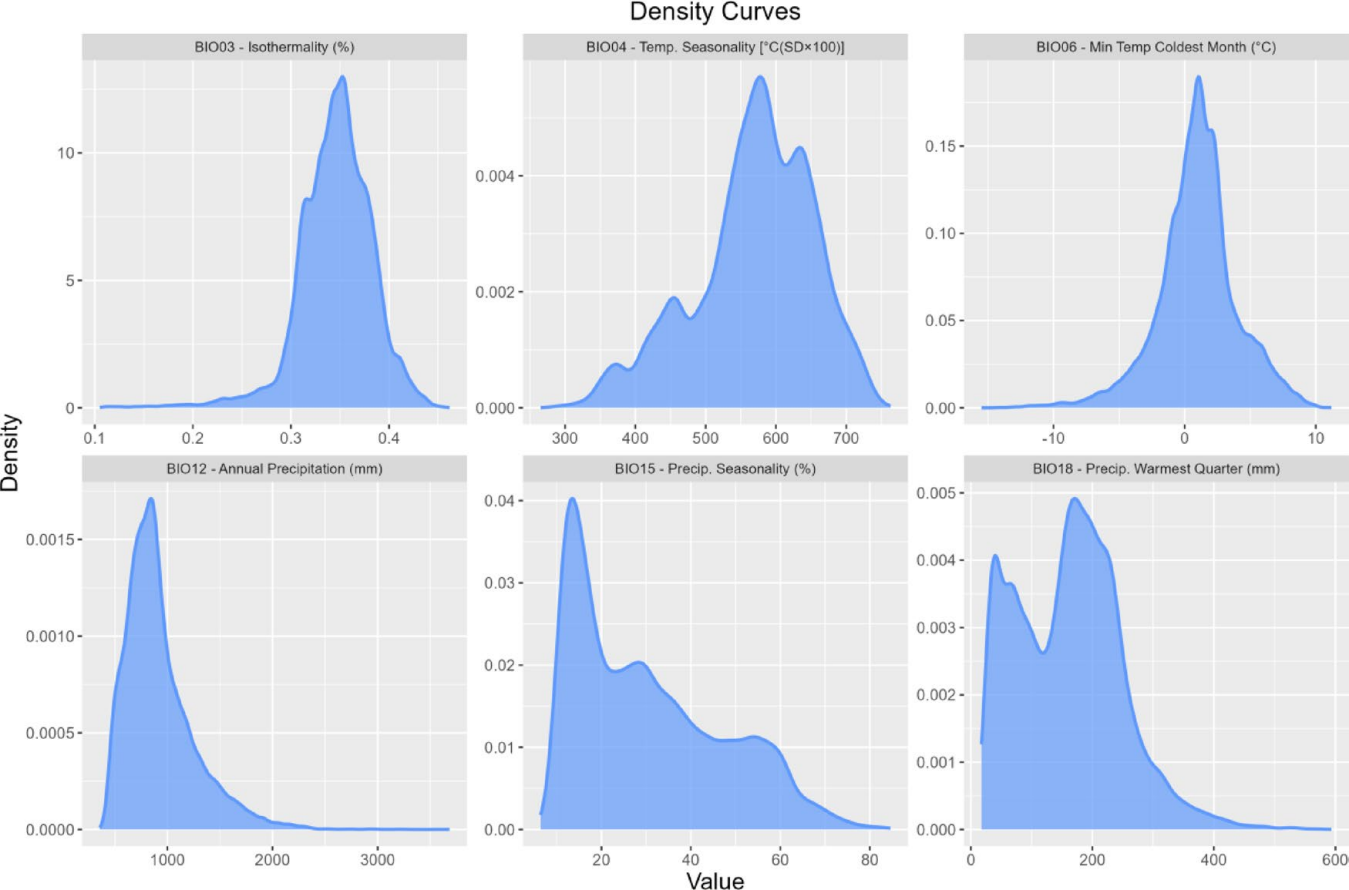
Density estimation for each selected environmental variable, including the 4 submediterranean oaks species used in the study: Bio03 (isothermality; %); Bio04 (temperature seasonality; °C(SD × 100)); Bio06 (minimum temperature of the coldest month; °C); Bio12 (annual precipitation; mm); Bio15 (precipitation seasonality; %); Bio18 (precipitation during the warmest quarter; mm).

The density estimation of each variable, considering the total occurrence points used in the model, can be seen in Fig. 1. The Bio03 values, lower than 0.5, suggest significant differences between seasons compared to daily temperature changes, but the Bio04 indicates a low temperature seasonality for most of the occurrences, showing that there are differences in temperature between the different seasons of the year, even though they are not heavily pronounced. The response curves for minimum temperature of the coldest month (Bio06) and temperature seasonality (Bio04) indicate cold winters and marked thermal seasonality, characteristic of seasonal climates found in transitional zones towards inland Mediterranean areas. Regarding annual precipitation (Bio12), values indicate a range between 500 mm and 1200 mm, with moderate seasonality (Bio15), indicating the existence of noticeable wet and dry periods, although not extreme. Some of this precipitation occurs in the summer, and few points have null precipitation values during the warmest quarter (Bio18) (Fig. 1).

### Current and future distribution of the submediterranean ecotone

**Fig. 2:**
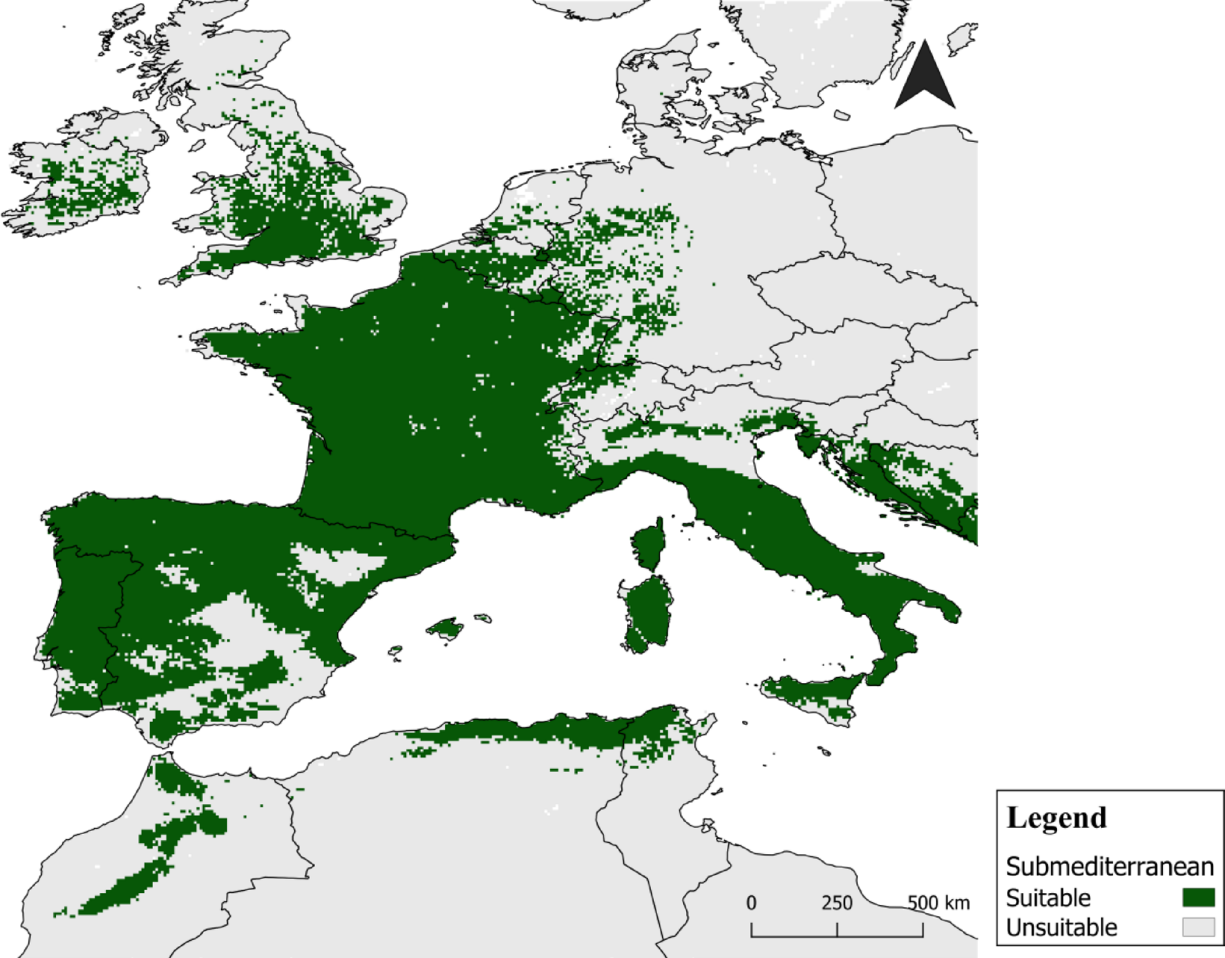
Modelled suitable distribution of the submediterranean ecotone in the western Mediterranean area under current climate conditions, based on marcescent oaks occurrence points (Q. *canariensis*,* Q. faginea*,* Q. pubescens* and Q. *pyrenaica*). Maps were generated by IP in R v.4.2.0 (https://www.r-project.org) and assembled in QGIS 3.34.6 (Spatial without Compromise · QGIS Web Site).

The final ensemble model suggests that under current climatic conditions the submediterranean ecotone spans into a significant portion of Western Europe, including Great Britain and Ireland, and extends into Northern Africa, from the coastal regions of Tunisia and Algeria to the Moroccan Atlantic Mountain ranges (Fig. 2).

**Fig. 3:**
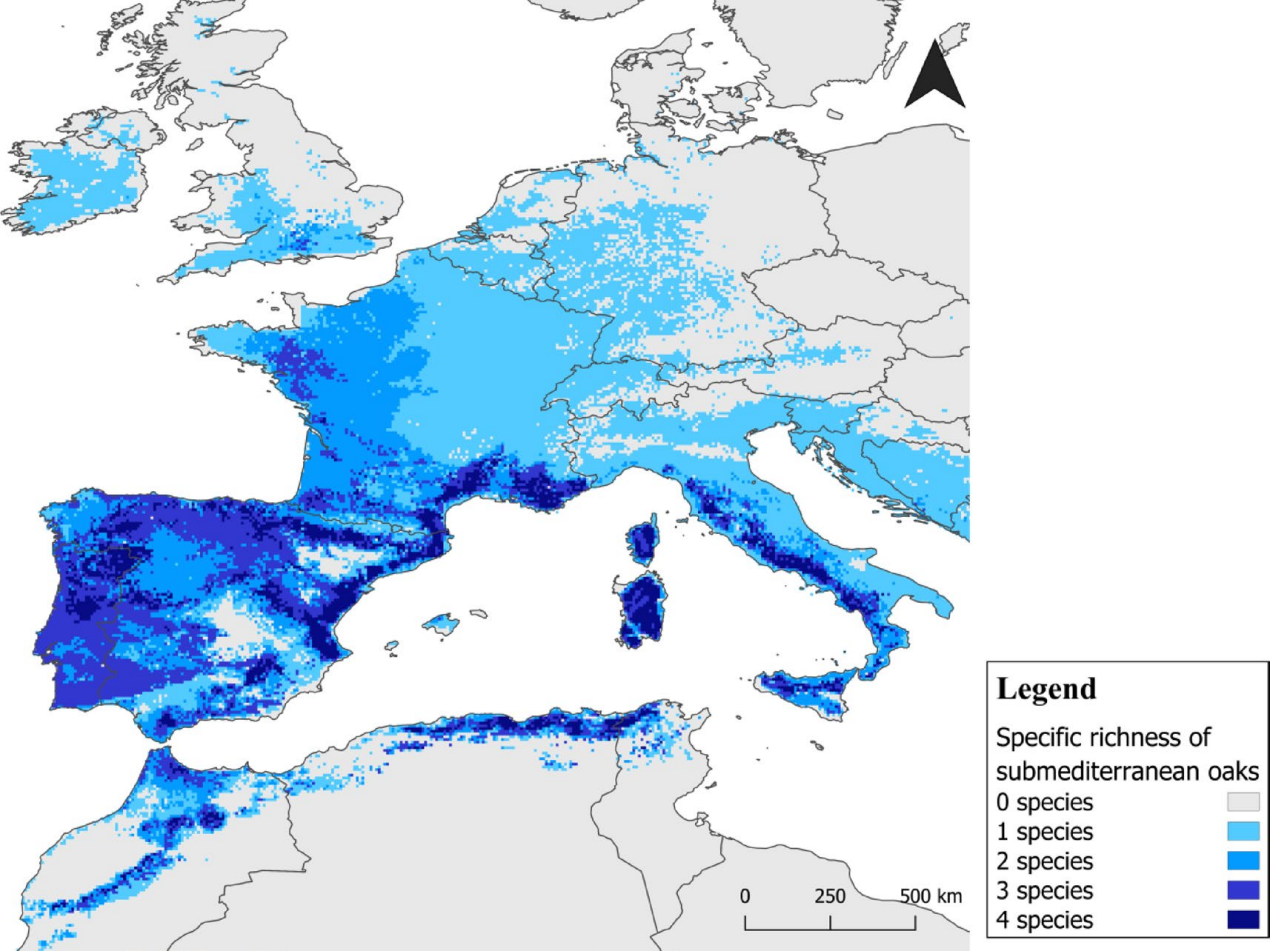
Species richness (number of species) of marcescent oak species in the submediterranean ecotone, suitable for the indicated area, given by overlapping (summing) the modelled suitable area for each species (*Q*. *canariensis*,* Q. faginea*,* Q. pubescens* and/or Q. *pyrenaica*) under current climate conditions. Maps were generated by IP in R v.4.2.0 (https://www.r-project.org) and assembled in QGIS 3.34.6 (Spatial without Compromise · QGIS Web Site).

In the southern part of the study area, including the Iberian Peninsula, Sardinia, Corsica, and North Africa, suitable environmental conditions seem to exist for all species, especially along the coastline. In the northern and eastern regions, appropriate conditions occur for one or two of the considered species (*Q. pubescens* and *Q. pyrenaica*). At the same time, some coastal areas have the potential for up to three marcescent species. In North and Eastern locations, conditions are suitable for only one species (*Q. pubescens*) (Fig. 3).

**Fig. 4:**
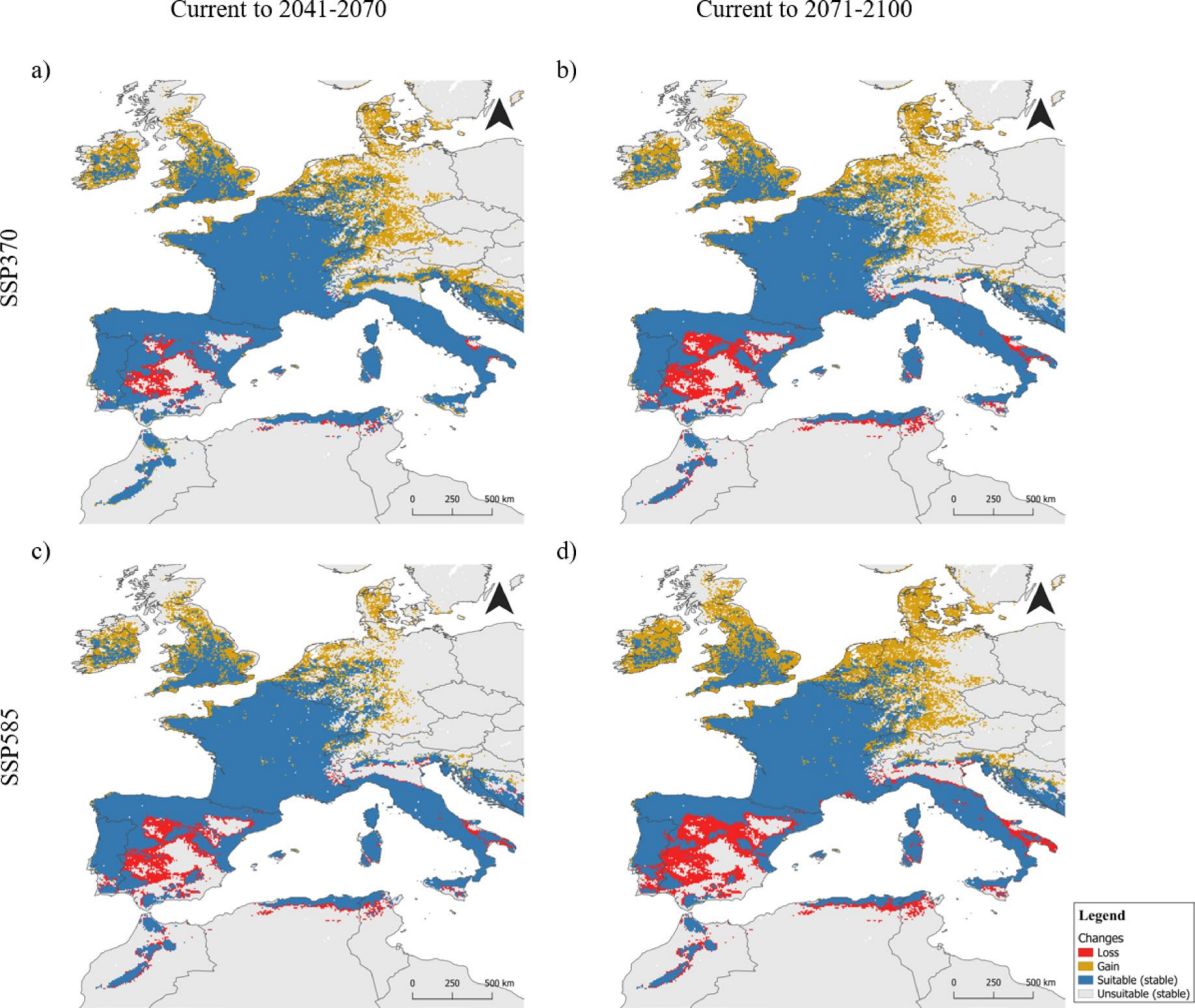
Changes in predicted distributions (gain = orange, lost = red, or stable = blue) of the submediterranean ecotone in the western Mediterranean area: (a) 2041–2070 SSP370; (b) 2071–2100 SSP370; (c) 2041–2070 SSP585; (d) 2071–2100 SSP585. Maps were generated by IP in R v.4.2.0 (https://www.r-project.org) and assembled in QGIS 3.34.6 (Spatial without Compromise · QGIS Web Site).

**Fig. 5:**
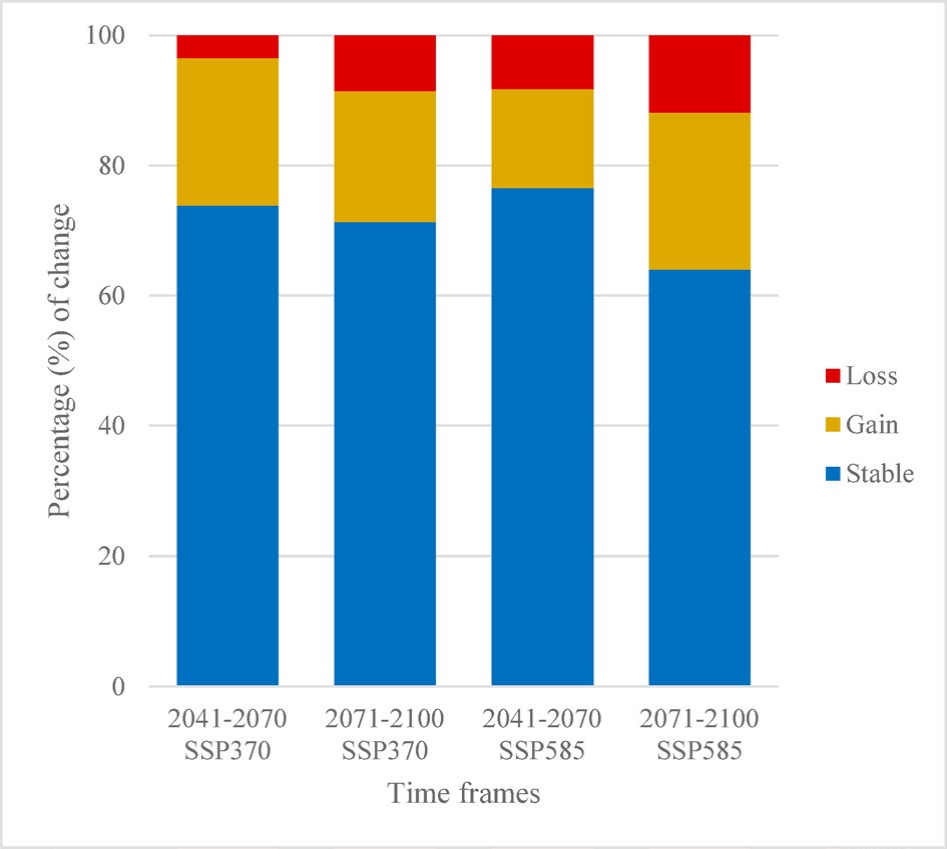
Percentage of change (% change) of the predicted distributions (gained = orange, lost = red, or stable = blue) of the submediterranean ecotone in the west Mediterranean area in the focal time frames.

Projections under future climatic scenarios indicate a northward (latitudinal) and eastward (longitudinal) shift in the geographic range of submediterranean conditions for the analyzed time-windows (2041–2070 and 2071–2100), and for the two different SSP scenarios. As anticipated, these shifts will be more pronounced under the most pessimistic scenario (SSP585) and in later periods (2071–2100) (Fig. 4). Expected gains will include northern and eastern Europe, with submediterranean ecotone potentially reaching as far as Scandinavia, particularly through the influence of *Q. pubescens* outposts. In contrast, significant losses are anticipated in southern areas, especially in North Africa inland regions and southern Iberia. However, coastal zones and higher altitudes in the southern regions are expected to remain suitable (Fig. 4). Overall, projections suggest that 60–80% (from 20,860 km^2^ to 22,327 km^2^) of the current submediterranean area will remain suitable across all scenarios considered. In most scenarios, the area gains are expected to exceed 20% (from 6244 km^2^ to 7865 km^2^), while total losses range from 3.5% (1127 km^2^) to 11.9% (3885 km^2^) (Fig. 5).

## Discussion

Our results show that the submediterranean ecotone spans a broad area, as inferred from the current distribution of marcescent oak species, used as a proxy. This ecotone intersects much of the Eurosiberian Region, and extends across the European Southern Peninsulas and into Northern Africa (Mediterranean Region). This result reflects the sum of the ecological preferences and reflects the ecological breadth of the four species used, which vary in their tolerance to drought and low temperatures^13^. Nonetheless, our results indicate that the distribution of these marcescent species is primarily influenced by annual precipitation, temperature seasonality, and minimum temperature of the coldest month. Higher annual rainfall values in drier Mediterranean areas are a key factor explaining spatial distributions of the selected oaks, including the Atlantic influence given by lower temperature seasonality, and the absence of extreme winter cold. Furthermore, the analysis of the results (Fig. 1) suggests that although there are well-defined wet and dry periods within a year, precipitation seasonality is not heavily pronounced, with precipitation occurring even in the drier period. Combined with moderate to high summer precipitation, this pattern reinforces water availability during the dry and hot season, which is a key driver that influences these submediterranean white oaks’ distributions, as shown by Vila-Viçosa et al.^11,27^.

Although the submediterranean ecotone spans a considerable portion of the Eurosiberian Region, not all areas appear suitable for all the considered species (Fig. 3). The Iberian Peninsula, along with Sardinia, Corsica, and the coastal regions of southern France, Italy, and North Africa, presents suitable conditions for two of these species. Most of these regions are suitable for at least three species, and in several locations, particularly in mountainous and coastal zones, suitable conditions exists for the four species. Additionally, parts of western France show suitability for two or three species. In contrast, the northern and Eastern areas are suitable for only one species: the downy oak (*Q. pubescens*).

The *Q. pubescens* retains significant morphological variability, shaped by a complex history of survival and migration events during the Pleistocene glaciations, as well as by population fragmentation caused by prolonged human impact^28^. Additionally, *Q. pubescens* occupies diverse ecological positions at the landscape level, allowing it to thrive in unfavored biotopes and secondary positions in areas dominated by deciduous temperate species like *Fagus sylvatica*, *Q. robur*, and *Q. petraea*^29^. In the other biogeographic extreme, *Q. pubescens* can survive in typical Mediterranean areas, contacting with evergreen oaks like *Q. rotundifolia*^30^ in submediterranean areas^31^. This species’ ecological plasticity is evident when analyzing the occurrences dispersion, considering the most impacting environmental variables, particularly the minimum temperature of the coldest month (Supplementary Fig. 3). The secondary positions and the broad bioclimatic niche of *Q. pubescens*, with its wide distribution across Europe, have led to an overestimation of the resulting model^28^particularly at the range edges, leading to the definition of areas as submediterranean, but where only this species has the conditions to persist. In contrast to *Q. faginea* and *Q. pyrenaica*^32,33^, *Q. pubescens* is expected to have its suitable habitat expanded in the coming years, particularly across central and northern Europe^31,34^. However, due to its preference for areas with lower precipitation seasonality and at least 150 mm of precipitation during the warmest quarter (Supplementary Fig. 2), the range of *Q. pubescens* is projected to contract at its southern limits^16^.

The pyrenean oak (*Q. pyrenaica*) is also often present among *Fagus sylvatica* and *Q. robur* forests^35^both in altitude and in northern latitudes. This species has a sharp Atlantic distribution and seems more adapted to dry summers and higher precipitation seasonality, but it can tolerate higher amounts of annual rainfall (Supplementary Fig. 2).

The oaks *Q. canariensis* and *Q. faginea* seem to be better adapted to lower precipitation amounts during the warmer months and prosper in places where minimum temperatures in the coldest month are above zero degrees Celsius. Still, there are occurrences of *Q. pubescens* and *Q. pyrenaica* that thrive in similar conditions (Supplementary Fig. 2), and are mostly associated with areas where more than three marcescent oak species meet suitable conditions. Although *Q. canariensis* and *Q. faginea* have been described as thermophilic taxa, inhabiting dry conditions^12^, the importance of occult precipitation, associated with summer fogs, and terrain ruggedness, which loses importance in higher scale models^36^, should not be overlooked as predictors of these species’ distributions^11^. In the case of *Q. canariensis*, increasingly drier conditions on inland areas since the Last Glacial Maximum acted as limited factor for its expansion, proving the importance of hidden precipitation on areas influenced by the Atlantic Ocean^11^. Indeed, this marcescent oak prefers fresh, well-drained soils low in carbonate materials, typically found in valleys and along streams and creeks. These habitats offer more humid microclimatic conditions, making the oak particularly sensitive to local environmental changes, especially in drier climates. It is associated with an upper sub-humid ombrotype, but can also inhabit upper dry areas in more sharply oceanic regions. Furthermore, it can only occur in more inland contexts where specific microclimatic conditions prevail. It requires the presence of low-intensity, often occult precipitation, combined with thermal inversion layers forming within deep valley systems. These conditions enhance the condensation of atmospheric water vapour, resulting in occult precipitation, particularly during the summer months^37^ (and references therein).

The dynamic nature of the submediterranean ecotone during the late Quaternary has been well-documented for the Iberian Peninsula^11^. Vila-Viçosa et al.^11^ identified key paleoclimatic drivers that shaped the historical dynamics of this ecotone, but our results suggest that future trajectories may follow contrasting pathways. For instance, species like *Q. pubescens* may expand into new bioclimatic zones, potentially reversing patterns of past contraction. This diachronic perspective reinforces the need for dynamic conservation planning that acknowledges both paleoecological legacies and future climate uncertainty. While the time intervals analyzed in our study — 60 and 90 years — are relatively short compared to those examining range shifts between past and present climates (Last Glacial Maximum - ca. 21 Ky and Mid-Holocene - ca. 6 Ky)^11^they still confirm the ongoing dynamism of this ecotone, probably because these changes are happening faster than those seen in the past. Our results show a range contraction in southern inland Europe and north Africa, with an altitudinal refugia in the Mediterranean mountains and an expansion into northern regions^31^contradicting previous studies, which predicted a decline in submediterranean regions with limited potential for expansion to other areas^13^. The results indicate a general northward shift for this ecotone, driven by the loss of suitable habitat at the southern rear edge of the distribution (Mediterranean areas) and an expansion at the northern front edge. The loss of suitable areas for marcescent oaks is marked in lower altitudes of the western Mediterranean area, with an expected turn-over to evergreen species like cork oak, round-leaf oak (*Q. suber* and *Q. rotundifolia*)^38^ and strawberry tree (*Arbutus unedo*)^39^. Southern areas with a sharper Atlantic influence will likely benefit from milder climatic conditions and local factors, such as summer fog, influenced by the moderating effects of the Atlantic Ocean or the Mediterranean Sea. These factors could compensate for reduced rainfall, allowing submediterranean forests to persist. As a result, these areas may become crucial refuges for southern marcescent oak populations and other species that inhabit these forests^11^. *Quercus canariensis*, as referred to above, is an example of a species confined to southern refugia after the last glaciation^40^and prone to be dramatically affected by global warming after deep human-induced fragmentation. On the opposite, the expansion of the submediterranean ecotone into northern regions^31^overlapping deciduous Eurosiberian forests^33^and possibly increasing species diversity and suitability in those areas^31,34^. Marcescent species emerge as fundamental for anticipating the suitability loss for deciduous and temperate species like *Q. robur*,* Q. petraea*, and even *Fagus sylvatica*^41^. In the front edge (northern margin), marcescent oaks often neighbor temperate and deciduous species, sharing many understory species^15,29,41–43^. Thus, they can function as a buffer against Ecosystem Services decline and loss of suitability for temperate and deciduous species, which are expected to contract and lose productivity in the southeastern areas of their range^44^. As climate change progresses, marcescent species are expected to gradually benefit from increased recruitment, while temperate species, more sensitive to drought-induced stress, are projected to experience higher mortality and decreased recruitment^41^ promoting species turnover. Similar dynamics are expected in the southern areas, where the submediterranean ecotone is increasingly constrained by rising temperatures, reduced annual precipitation, and shifting spatial climatic patterns. These shifting patterns involve not only changes in mean temperature and precipitation levels, but also seasonal adjustments on time and spatial patterns, with higher intensity and frequency of climatic extremes, such as heat waves, droughts, and heavy rain events^45^. In these regions, Mediterranean evergreen oaks, better adapted to prolonged drought, are likely to gain a competitive advantage, gradually outcompeting marcescent species, specially in mixed forests where both occur^38^.

It is expected that the effects of climate change will first be noticed in areas with less favorable conditions for the present species or degraded forest stands, such as growth and productivity reduction^44^ or increased mortality^32^. Therefore, focusing on adapting forests gradually to future projected conditions may be the key^46^ to avoid massive forest decay in the upcoming years in submediterranean areas.

The results of our study clearly show that the submediterranean ecotone is shifting northward with rising temperatures and decrease in precipitation due to climate change. While it is likely to maintain or expand its range, submediterranean forest compositions may be modified. Further research is needed to assess the future trajectories of individual species, as some may experience a reduction in suitable habitats and face challenges in migrating to new areas, such as *Q. canariensis*, with a Data Deficient and unknown IUCN status^47^. This information will be crucial for planning effective present and future management and conservation strategies.

Adaptive forest management and restoration are critical for preserving these forests^41^particularly at their southern range margins, but also to mitigate Ecosystem Services loss in northern areas, where temperate forests may be at risk. These efforts must adopt a long-term perspective, as they aim to prepare marcescent forests to withstand future climate conditions over several decades^6^. Strategies could focus on enhancing species diversity and forests drought tolerance. Tree species’ genetic diversity enhances forest resistance against natural hazards and facilitates survival in the seedling stage, especially in drier areas^48^and is a key factor in preserving biodiversity associated with forests^49^ and Ecosystem Services provisioning^50^. It is also important to increase the resistance and resilience of northern populations and incorporate individuals from populations in less suitable areas, and thus more drought-resistant^44,51^. This approach, known as assisted migration, may also involve introducing new species into transition zones^51^or relocating species to projected favorable areas where natural dispersal is limited or unlikely^23^. To operationalize these strategies, it is key to identify possible source and destination areas and ensure that the latter remain stable over time^34^. To accomplish this, it is crucial to increase the detail of single-species models, since large-scale models may not capture the importance of edaphic and topographic variables, which play a relevant role in environmental heterogeneity^36^. Further studies are needed to develope species-specific models at a finer spatial scale for the present and also for the future, considering global warming scenarios to help conservation programs design, to alert policy makers, and to increase mitigation efforts.

## Conclusions

Our study addresses a crucial gap in southern European vegetation research by examining the current and future extent of the submediterranean ecotone, a key bioclimatic and biogeographic zone influencing plant and habitat diversity across Europe and North Africa. Our findings reveal an expansion in the suitability of marcescent species toward northern regions, while southern areas are projected to contract, with the most significant losses occurring in the interior of the Iberian Peninsula. In these southern regions, the submediterranean ecotone is expected to persist at higher altitudes or along coastal areas, where the buffering effect of the sea, particularly in terms of lower temperature ranges and higher precipitation, will support its maintenance. This research provides valuable insights for developing new forest management strategies aimed at ecosystem preservation and restoration, biodiversity conservation, carbon allocation, and supporting the European Commission’s Nature Restoration Law proposal. Future studies should focus on endangered species, incorporating meta-analyses that could offer a broader understanding of other species beyond oaks and trees.

By bridging projections from the past and the future, this study highlights the long-term ecological dynamism of the submediterranean ecotone. Understanding these temporal transitions is critical for anticipating climate-driven species redistribution and for devising conservation strategies that are robust to historical contingencies and future uncertainties.

## Methods

### Study area

**Fig. 6:**
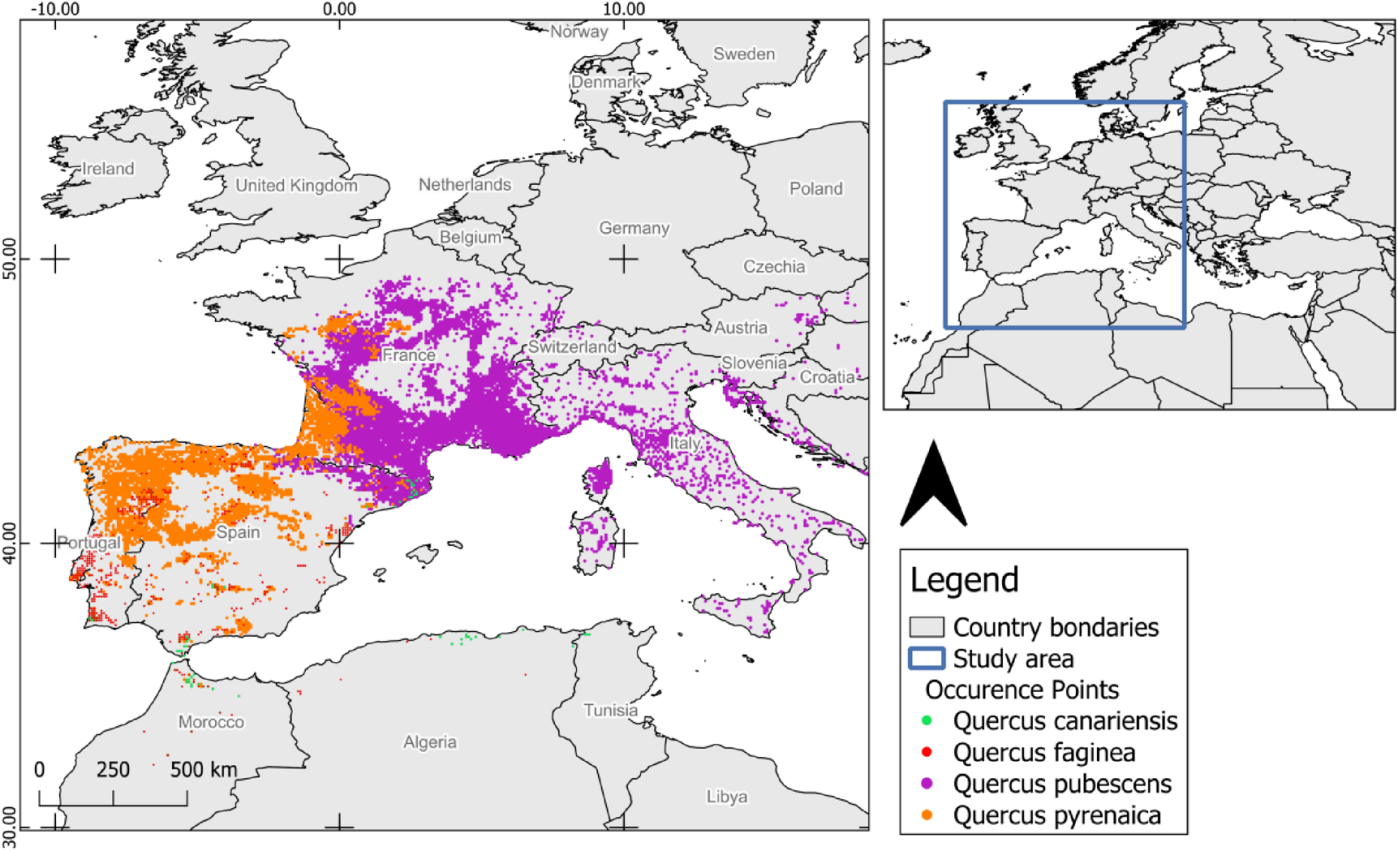
Study area and occurrence points of marcescent oak species (*Q*. *canariensis*,* Q. faginea*,* Q. pubescens* and *Q*. *pyrenaica*). Latitude/longitude coordinates in WGS 1984 (EPSG:4326) coordinate reference system grid. Maps were generated by IP in QGIS 3.34.6 (Spatial without Compromise · QGIS Web Site).

The study area encompasses the western Mediterranean Basin and a large portion of Western Europe, covering a total area of approximately 7.7 × 10^6^ km² (Fig. 6), holding a highly diverse geology. It is divided into two biogeographic regions: the Eurosiberian and the Mediterranean^10^. The southern part of the area is dominated by a Mediterranean climate, while the northern part experiences a temperate climate^8^. A submediterranean ecotone can be identified in the transition between those two macroclimates.

### Focal species and occurrence data

We selected four marcescent oaks that dominate submediterranean forests in the Iberian Peninsula: *Q. canariensis*, *Q. faginea*, *Q. pubescens*, and *Q. pyrenaica*. Their distribution also extends to other parts of Europe and North Africa. (see Supplementary Table 1 for focal species description).

Species occurrence data were gathered from multiple sources. Records for all species were compiled from online databases including expert-curated knowledge, particularly for the Iberian Peninsula, and Vila-Viçosa et al.^11,27^.

Species records (presence-only data) were aggregated in a database using ArcGIS Pro^®^ 3.2.2. The data were filtered and occurrences with georeferencing errors, and duplicated records were removed. The resulting dataset comprehends 84 occurrences of *Q. canariensis*, 509 occurrences of *Q. faginea*, 5247 occurrences of *Q. pubescens*, and 2833 occurrences of *Q. pyrenaica* (Supplementary Table 3). All records were then consolidated and harmonized into a 10 km × 10 km grid, resulting in 8342 occurrences, with latitude/longitude coordinates in the WGS 1984 (EPSG:4326) coordinate reference system grid (Supplementary Table 4).

Additionally, to verify whether the entire submediterranean area would be equally suitable for all species, an individual suitability model for the current time was created for each species. The species’ suitability models were then overlapped, creating a spatial oak richness map in the submediterranean ecotone.

### Environmental variables

A total of 19 bioclimatic indices were sourced from the CHELSA v.2.1 dataset^52^ for the current calibration period 1981–2010 and two future periods (2041–2070 and 2071–2100). The future climate projections were derived from averaged ensemble projections for five General Circulation Models (GCMs) (GFDL-ESM4, IPSL-CM6A-LR, MPI-ESM1-2-HR, MRI-ESM2-0, and UKESM1-0-LL) for the Coupled Model Intercomparison Project Phase 6 (CMIP-6), considering two different Shared Socioeconomic Pathways (SSP) scenarios, SSP370 (medium scenario) and SSP585 (pessimistic scenario), and two future periods (2041–2070 and 2071–2100). We selected those five GCMs, since they are classified as having good performance (‘Satisfactory’) for the European region based on the previous CMIP5 Project^53^. regarding the SSP, we opted for two different scenarios, an intermediate scenario and a pessimistic scenario. Although the choice of a pessimistic scenario may not always be consensual, we chose to use both scenarios to capture the range of potential outcomes. This approach enables us to anticipate likely trends, inform conservation and mitigation strategies, and draw the attention of policymakers. All bioclimatic data were later re-projected and re-sampled (average) to a common reference grid at 10 km × 10 km spatial resolution (WGS 1984 (EPSG:4326)).

Additionally, we incorporated data on soil characteristics and terrain morphology^29^since these may be important variables to some of the considered species^11,27^including soil pH data and soil texture class at 5 cm depth^54^Topographic Ruggedness Index^55^ (TRI; as a proxy of slope and terrain complexity), retrieved from the EarthEnv project (Global 1,5,10,100-km Topography - EarthEnv), and Topographic Wetness Index (TWI; as a proxy of soil moisture and flow accumulation)^56^ derived from the EarthEnv project elevation data at 1 km spatial resolution.

The variable selection for each species model excluded collinear predictors (pairwise correlation |*r*| < 0.7)^57^, and further considered known species’ ecological requirements.

### Modelling approach: calibration, fitting and evaluation

SDMs were developed using the biomod2 package^58,59^ implemented in R statistical software (R 4.2.0)^60^. This package applies a multi-model ensemble forecasting approach by combining several statistical and machine-learning-based algorithms, to analyze species-environment relations^58,59^. Models were fitted using eight available modeling techniques currently available in biomod2: GLM (Generalized Linear Models); GAM (Generalized Additive Models); SRE (Surface Range Envelope); ANN (Artificial Neural Networks); FDA (Flexible Discriminant Analysis); MARS (Multivariate Adaptive Regression Splines); RF (Random Forests); and MAXENT (Maximum Entropy Model).

To avoid spatial niche truncation and clamping effects^61^models were developed over a significantly larger area encompassing the entire distribution range of the selected species with a buffer of 1000 km.

Default hyperparameters were used for all modelling techniques, except for the smoothing degree term in GAM algorithm, which was set to k = 4, and the number of boosting trees in GBM (n.trees = 2500), which were adjusted to minimize overfitting^62^.

Given the presence-only nature of the data, pseudo-absence (PA) datasets were generated for model calibration. Five PA sets of 10,000 grid cells were created without minimum distance between pseudo-absences, to maximize the representativeness of the study area’s environmental space. Species prevalence was adjusted to *p* = 0.3, which optimized model performance.

Holdout cross-validation was employed for model evaluation, with 80% of the input records used for model fitting and 20% for model evaluation at each round. A total of 10 rounds were performed for model evaluation.

For model performance assessment, the Area Under the Receiver-Operating Curve (AUC), True Skill Statistic (TSS), and Sensitivity and Specificity values were calculated in biomod2 ^60^. The AUC values vary between 0 and 1, and can be classified as excellent (0.9–1.0), very good (0.8–0.9), good (0.7–0.8), fair (0.6–0.7), and poor (0.5–0.6)^63^. The TSS can assume values between − 1 and 1, and can be classified as excellent (TSS > 0.8), good (0.6–0.8), fair (0.4–0.6), poor (0.4–0.2) and, very-poor (TSS ≤ 0.2)^64^. We used the threshold value maximizing the TSS^65^ to ‘binarize’ projections into suitable/unsuitable habitat.

To reduce inter-model uncertainty, we calculated an ensemble model based on an average of the best-performing models^26^. For selecting the best-performing models, a threshold of 0.7, based on TSS values was used^66^.

Variable importance was assessed using biomod2’s internal method through a permutation approach. A value of zero assumes no influence of a given variable, and a higher score indicates a greater influence of a variable in the model predictions.

The model representing current conditions was used as a baseline to calculate present-to-future range shifts. The current model overlapped with the different future model projections to assess range shifts, suitability gains/losses, and areas that remain suitable or unsuitable (Table 2). These operations were made in ArcGIS Pro^®^ 3.2.2, using the Raster Calculator tool.

**Table 2:** Matrix displaying species distribution dynamics by comparing current and future projections.

		Future model projections	
		**Unsuitable**	**Suitable**
Current projection (baseline)	**Unsuitable**	Stable unsuitable ($\:{S}_{u}$)	Gain ($\:G$)
	**Suitable**	Loss ($\:L$)	Stable suitable ($\:{Ss}_{}$)

A many-to-many matrix was assembled by combining all possible present-to-future comparisons for each species, allowing future variation to be visualized on maps.

## Electronic supplementary material

Below is the link to the electronic supplementary material.

Supplementary Material 1

Supplementary Material 2

## Data Availability

The authors declare compliance with Scientific Reports’ policy regarding data availability. All relevant data are available within the paper and its Supplementary Information files. Any other information about this paper can be given to the corresponding author upon request.
