# Supplementary material for: Tracking submediterranean ecotone shifts under climate change scenarios using marcescent oaks as indicators
Source: Sci Rep. 2025 Nov 10;15:39201. doi: 10.1038/s41598-025-10088-w (PMC12603024; doi:10.1038/s41598-025-10088-w)
Supplement: Supplementary file 2 — Supplementary Material 2 [file 41598_2025_10088_MOESM2_ESM.docx]

Supplementary Material

**Supplementary Table 1.** Focal species description

| **Focal Species** |
| --- |
| The Algerian oak (*Quercus canariensis* Willd.) is a tree that can reach up to 30 meters in height ^1,2^. Its distribution ranges from the north of Spain to Portugal, Morocco, Algeria, and Tunisia. In the Iberian Peninsula, it is considered it is considered a relict species, with a fragmented distribution ^1,3^, consisting of five small subpopulations ^4–6^ . It is classified as Critically Endangered in Portugal ^7^.In Europe, subpopulations are exclusive from siliceous bedrock, while in North Africa, they grow in limestone ^8^. The species is found at altitudes ranging from near sea level (100 m) to a maximum of 1,600 meters in the Atlas Mountains ^1^. It thrives in areas with annual rainfall ranging from 600 to 1,000 mm and prefers northern slopes, areas with higher soil humidity, or locations with frequent summer precipitation, such as advection fogs or thermal inversions ^1,4,5,8^, a key factor explaining its distribution ^4^. Average annual temperatures range from 12°C to 16°C, with the coldest and hottest months averaging 0°C and 24°C, respectively ^1^. |
| The Portuguese oak (*Quercus faginea* Lam.) is a mid-size slow-growing tree that typically does not exceed 20 meters in height ^1^. The species exhibits considerable morphological plasticity, leading to ongoing discussions about its taxonomy and nomenclature ^1,9–11^. Recently, the species was clarified to encompass two subspecies, previously considered separate taxa: *Q. broteroi* and *Q. faginea* ^8^. The distribution area encompasses the western IP (Portugal and Spain) and North Africa (Morocco, Algeria, and Tunisia ^1,8^, with additional populations in southern France and Mallorca ^1,12^. This oak can withstand temperatures ranging from 22°C, on average, in summer to -5°C in winter ^1^, and it can be found at altitudes from sea level to 1,900 meters. It is a drought-adapted species, thriving in areas with as little as 500 mm of annual precipitation ^11^, although it can tolerate rainfall as low as 250 mm, provided at least 100 mm occurs during summer ^1^. Summer precipitation is a key factor in its distribution ^4^. It typically inhabits sub-humid to humid ombrotypes, or more temporary, hygrophilous sites in drier areas ^8^. The species does not have a specific lithological preference ^8^, but it is often found on basic soils such as limestone, as it is displaced from acidic soils by *Quercus pyrenaica* ^1^. |
| The Pyrenean oak (*Quercus pyrenaica* Willd.), is an emblematic species of the IP forests, with a wide potential distribution range that includes South-western France, Spain, Portugal, and North-eastern Morocco ^13–15^, including important relictual subpopulations in mountain areas of the Mediterranean half of the IP and Morocco. It usually forms closed forests, although the level of closure can vary depending on management practices ^13,14^. This species can tolerate a high range of conditions, colonizing areas with average annual precipitation ranging from 600 mm to over 3000 mm, from which 100 to 200 mm must fall during the summer ^1^. It is found in areas with average annual temperatures between 6 and 15 ºC. ^15^, but it withstands temperatures from an average of -5 ºC in winter to 22 ºC in summer ^1^. Summer drought is a limiting factor ^1,6,15^, though the species has adapted morphologically and physiologically to tolerate moderate drought ^15^, exhibiting marcescent behavior in thermophilic areas ^8^. *Quercus pyrenaica* occupies a significant altitudinal range, from 290 m in the Basque country to 2,000 meters in the Rif mountains ^1,15^. Dryness in the Mediterranean region makes lower altitudes unsuitable for the species, so it is mostly found in mountain ranges typically above 1000 meters ^15^. It thrives in ombrotypes ranging from sub-humid to hyper-humid ^8^, and thermotypes from termo-mediterranean to supra-temperate belts ^15,16^. It generally prefers acidic lithologies, including siliceous soils such as granite, slate, schist, and gneiss ^1^. However, locally it can also grow in decarbonated basic substrates or neutral, due to the leaching of the carbonate existing in the soils ^17^. Vila-Viçosa et al. ^8^ classify the species as edaphic indifferent, although preferring siliceous bedrock. |
| The Downy oak (*Quercus pubescens* Willd.) typically grows up to 15-20 m. It is widely distributed in central and southern Europe, from western Spain to Ukraine and Anatolia, including European Mediterranean coastlines to central France, with populations on islands like Corsica, Sardinia, and Sicily ^18^. It grows under submediterranean and temperate climatic conditions and is considered the typical species of the submediterranean band in southern Europe ^1,17,19^. The species exhibits significant ecological plasticity, occupying areas from sea level to altitudes of 1,200-1,300 meters, and sometimes even up to 1,900 meters. It can tolerate moderate summer drought ^20^, but requires a rainfall of 600mm annually, ideally evenly distributed throughout the year ^1^. In drier regions, it is often found at higher altitudes ^1^. *Quercus pubescens* tolerates low temperatures during the winter but avoids areas with frequent frost and drought events, and high temperature amplitudes ^17,21^. This species is indifferent to soil pH, but it seems to prefer basic and neutral soils ^1^, being better adapted to calcareous soil than to acidic ones ^20^. Even though, some authors refer that it prefers acidic soils in southern warmer countries (e.g., Sicily and Crete), and calcareous soil in the northern area of its distribution ^17^. |

**Supplementary Table 2.** Geographic records (Coordinate System: Datum WGS1984 / UTM 30 N) (EPSG: 32630) aggregated into 10x10 km.

**Supplementary Table 3.** Original set of the 19 bioclimatic variables that were retrieved for modeling. The selected variables to use for modelling are displayed in bold.

| Variable | Code | Unit |
| --- | --- | --- |
| Annual mean temperature | BIO1 | °C * 10 |
| Mean diurnal range (mean of monthly (max temp - min temp)) | BIO2 | °C * 10 |
| **Isothermality (BIO2/BIO7) (* 100)** | **BIO3** | **%** |
| **Temperature seasonality (standard deviation *100)** | **BIO4** | **-** |
| Max. temperature of warmest month | BIO5 | °C * 10 |
| **Min. Temperature of Coldest Month** | **BIO6** | **°C * 10** |
| Temperature annual range (BIO5-BIO6) | BIO7 | °C * 10 |
| Mean temperature of wettest quarter | BIO8 | °C * 10 |
| Mean temperature of driest quarter | BIO9 | °C * 10 |
| Mean temperature of warmest quarter | BIO10 | °C * 10 |
| Mean temperature of coldest quarter | BIO11 | °C * 10 |
| **Annual precipitation** | **BIO12** | **mm** |
| Precipitation of wettest month | BIO13 | mm |
| Precipitation of driest month | BIO14 | mm |
| **Precipitation seasonality (coefficient of variation)** | **BIO15** | **%** |
| Precipitation of wettest quarter | BIO16 | mm |
| Precipitation of driest quarter | BIO17 | mm |
| **Precipitation of warmest quarter** | **BIO18** | **mm** |
| Precipitation of coldest quarter | BIO19 | mm |
| **Topographic Ruggedness Index** | **TRI** | **m** |
| **Topographic Wetness Index** | **TWI** | **-** |
| **Soil pH data at 5 cm depth** | **PH** | **H_2_O and KCl solution** |
| **Soil texture class at 5 cm depth** | **TEX** | **weight %** |

**
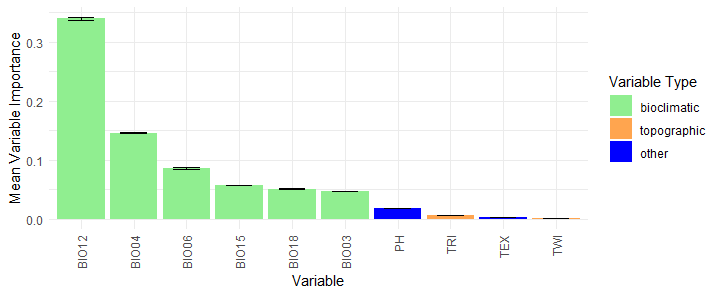
**

**Supplementary Figure 1.** Variable importance (average standard deviation across all models) for each oak species considering the three groups of predictors, climatic (precipitation and temperature), topographic, and others. Bio03— Isothermality (Bio02/Bio07) (×100); Bio04—temperature seasonality; Bio06—minimum temperature of the coldest month; Bio12—annual precipitation; Bio15— Precipitation Seasonality (Coefficient of Variation); Bio18—precipitation of warmest quarter; pH (soil pH at 5cm); TEX (soil texture class at 5cm); TRI (Topographic Ruggedness Index) and TWI (Topographic Wetness Index).

a)


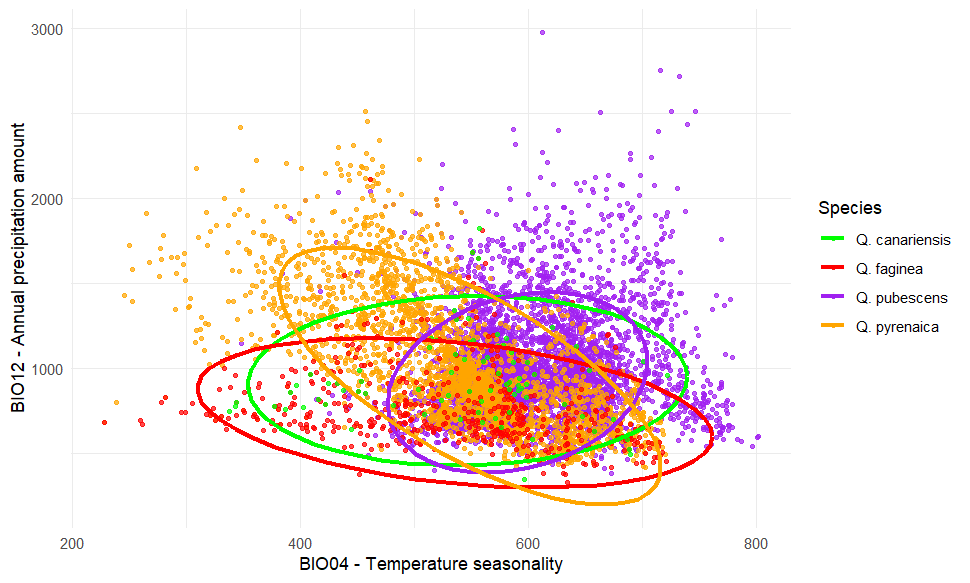


b)


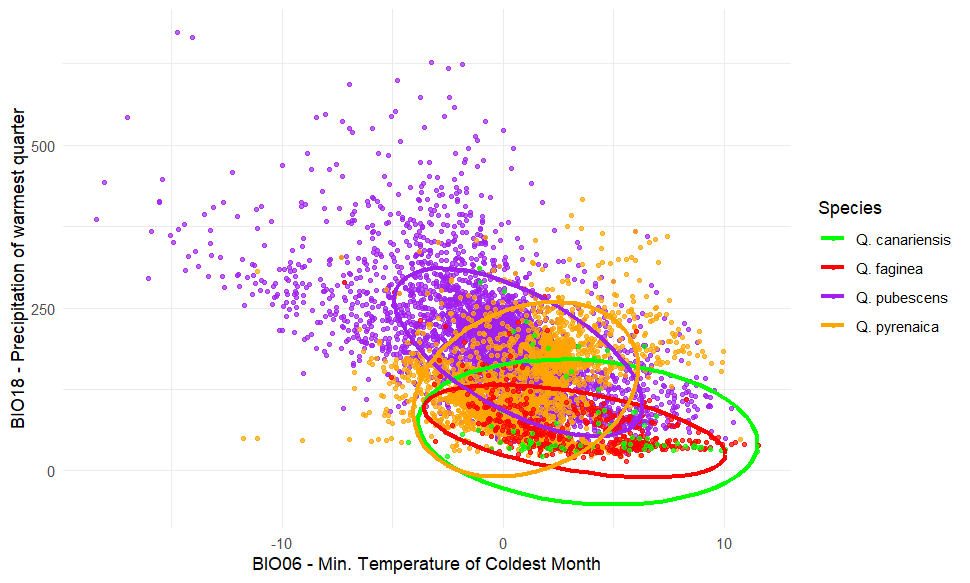


**Supplementary Figure 2.** Climatic preferences of the considered species, with a 95% confidence ellipse, considering the a) Temperature seasonality (Bio04) and the Annual precipitation amount (Bio12); b) Minimum Temperature of the Coldest Month (Bio06) and the Precipitation of the Warmest Quarter (Bio18) values for each presence point.


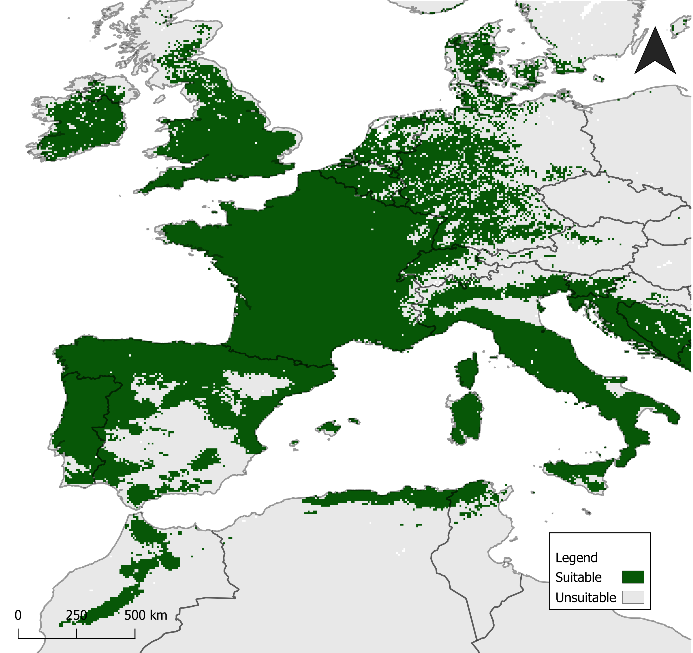

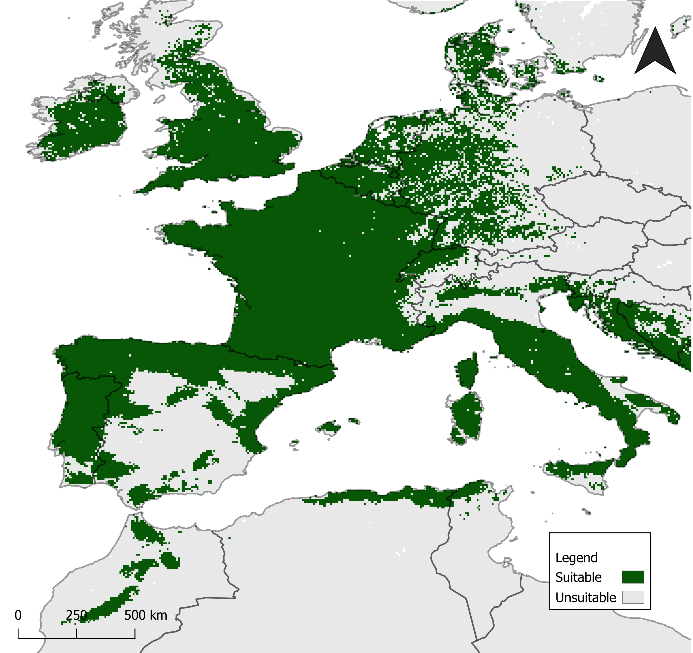

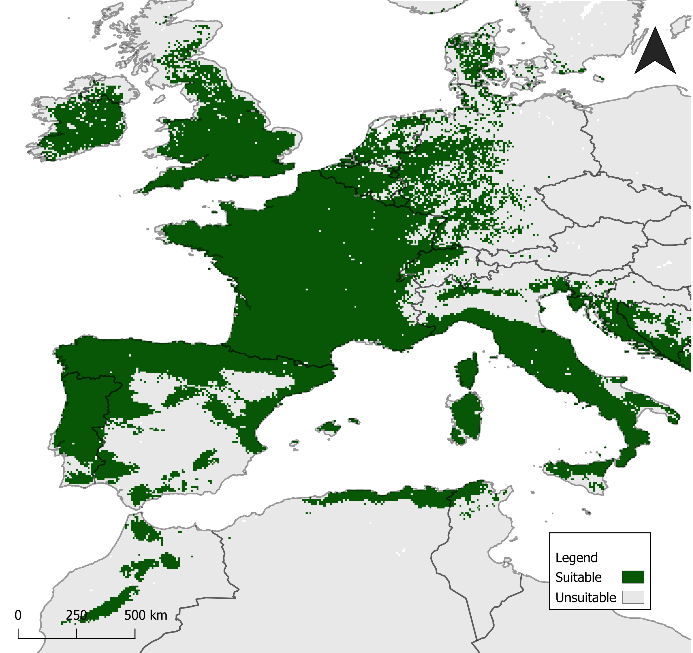

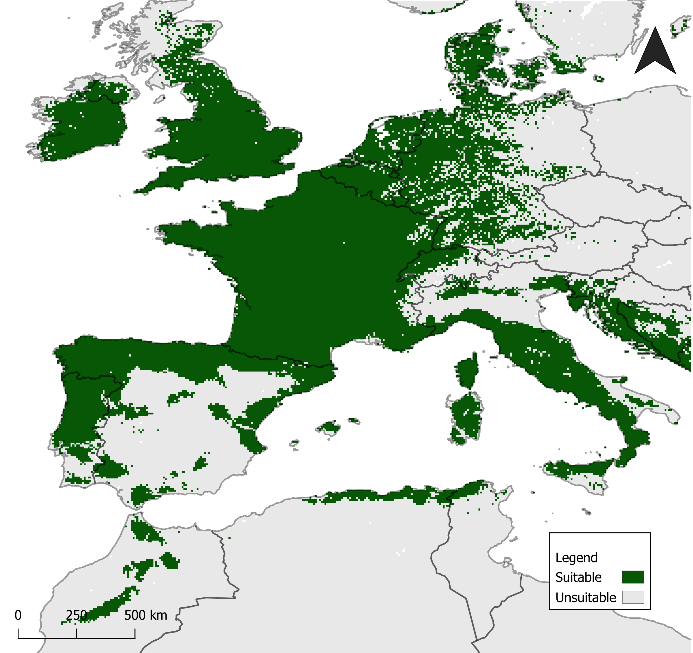


a)

c)

b)

d)

SSP370

SSP585

2041-2070

2071-2100

**Supplementary Figure 3.** Binary maps of projections for the future: presence (dark green)/absence (gray). (a) RCP 4.5 2041-2070, (b) RCP 4.5 2071-2100, (c) RCP 8.5 2041-2070, (d) RCP8.5 2071-2100. Maps were generated by IP in R v.4.2.0 (https ://www.r-proje ct.org) and assembled in QGIS 3.34.6 ([Spatial without Compromise · QGIS Web Site](https://qgis.org/)).

**References**

1. Castro, E. *et al.* *Los Bosques Ibéricos*. (Editorial Planeta, Barcelona, 2005).

2. Costa, T. M. O. Quercus canariensis facing climate change: Genomics and niche modelling as tool for conservation Orientador. (Universidade do Porto, Porto, 2020).

3. López, J. M. , Jiménez, J. G. & Camacho, C. A. Fitoclimatologia de Quercus canariensis Willd. en España. Potencialidades y Adecuaciones Fitoclimáticas. *Flora Montiberica* **29**, 14–19 (2005).

4. Vila-Viçosa, C. *et al.* Late Quaternary range shifts of marcescent oaks unveil the dynamics of a major biogeographic transition in southern Europe. *Sci Rep* **10**, (2020).

5. Vila-Viçosa, C. *et al.* Syntaxonomic update on the relict groves of Mirbeck’s oak (Quercus canariensis Willd. and Q. marianica C. Vicioso) in southern Iberia. *Plant Biosyst* **149**, 512–526 (2015).

6. Vila-Viçosa, C. *et al.* Combining satellite remote sensing and climate data in species distribution models to improve the conservation of iberian white oaks (Quercus l.). *ISPRS Int J Geoinf* **9**, (2020).

7. Carapeto, A., Francisco, A., Pereira, P. & Porto, M. *Lista Vermelha Da Flora Vascular de Portugal Continental.* vol. 7 (Imprensa Nacional, Lisboa, 2020).

8. Vila-Viçosa, C. M., Capelo, J. H., Alves, P., Almeida, R. S. & Vázquez, F. M. New annotated checklist of the Portuguese oaks (Quercus L., Fagaceae). *Mediterranean Botany* **Online first**, 1–46 (2022).

9. Aissi, A. New insights about Quercus faginea (s.l.) taxonomic status in northern Africa. *Mediterranean Botany* **44**, (2023).

10. Vázquez, F. M. *et al.* Anotaciones a la nomenclatura del género Quercus L. (FAGACEAE), en la Península Ibérica y NW de África. *Folha Botanica Extremadurensis* **12**, 5–79 (2018).

11. Ribeiro, H., Ferreira Tomás, J., Gonçalo Soutinho, J. & Martins Vila-Viçosa, C. *Old-Growth Quercus Faginea in Portugal*. https://www.researchgate.net/publication/361503906 (2022).

12. Alonso-Crespo, I. M. *et al.* Effect of the mother tree age and acorn weight in the regenerative characteristics of Quercus faginea. *Eur J For Res* **139**, 513–523 (2020).

13. Carvalho, J. P. *et al.* *O Carvalho Negral*. (Universidade de Trás-os-Montes e Alto Douro-CEGE, Vila Real, 2005).

14. de la Serna, B. V., Sánchez-Mata, D. & Gavilán, R. G. Marcescent Quercus pyrenaica forest on the Iberian Peninsula. in *Geobotany Studies* 257–283 (Springer, 2016). doi:10.1007/978-3-319-21452-8_10.

15. de la Serna, B. V. Comprehensive study of Quercus pyrenaica willd. forests at Iberian Peninsula: indicator species, bioclimatic, and syntaxonomical characteristics. (Universidad Complutense de Madrid, Madrid, 2014).

16. Pinto-Gomes, C., Paiva-Ferreira, R. & Meireles, C. New Proposals on Portuguese Vegetation. *Lazaroa* **28**, 67–77 (2007).

17. Salvatore, P., de Rigo, D. & Caudullo, G. Quercus pubescens in Europe: distribution, habitat, usage and threats. in *European Atlas of Forest Tree Species* (eds. San-Miguel-Ayanz, J., de Rigo, D., Caudullo, G., Durrant, T. H. & Mauri, A.) 156–157 (Publication Office of the European Union, 2016). doi:10.2760/776635.

18. Pasta, S., De Rigo, D. & Caudullo, G. Quercus pubescens in Europe: distribution, habitat, usage and threats. in *European Atlas of forest tree species* 156–157 (2016).

19. Wellstein, C. & Spada, F. The status of quercus pubescens willd. in Europe. in *Geobotany Studies* 153–163 (Springer, 2015). doi:10.1007/978-3-319-01261-2_8.

20. Contran, N. *et al.* Physiological and biochemical responses of Quercus pubescens to air warming and drought on acidic and calcareous soils. *Plant Biol* **15**, 157–168 (2013).

21. Mevy, J. P. *et al.* Response of downy oak (Quercus pubescens willd.) to climate change: Transcriptome assembly, differential gene analysis and targeted metabolomics. *Plants* **9**, 1–20 (2020).
